# The Role of SIRT3 in the Osteoporosis

**DOI:** 10.3389/fendo.2022.893678

**Published:** 2022-05-25

**Authors:** Siwang Hu, Shuangshuang Wang

**Affiliations:** ^1^ The Orthopaedic Center, Wenling First People’s Hospital (The Affiliated Wenling Hospital of Wenzhou Medical University), Wenling, China; ^2^ Department of Cardiology, Wenling First People’s Hospital (The Affiliated Wenling Hospital of Wenzhou Medical University), Wenling, China

**Keywords:** SIRT3, osteoporosis, bone formation, bone resorption, mitochondria

## Abstract

SIRT3 is an NAD^+^-dependent deacetylase in the mitochondria with an extensive ability to regulate mitochondrial morphology and function. It has been reported that SIRT3 participates in the occurrence and development of many aging-related diseases. Osteoporosis is a common aging-related disease characterized by decreased bone mass and fragility fractures, which has caused a huge burden on society. Current research shows that SIRT3 is involved in the physiological processes of senescence of bone marrow mesenchymal stem cells (BMSCs), differentiation of BMSCs and osteoclasts. However, the specific effects and mechanisms of SIRT3 in osteoporosis are not clear. In the current review, we elaborated on the physiological functions of SIRT3, the cell types involved in bone remodeling, and the role of SIRT3 in osteoporosis. Furthermore, it also provided a theoretical basis for SIRT3 as a therapeutic target for osteoporosis.

## Introduction

Osteoporosis is a systemic bone disease characterized by bone loss and bone tissue microstructure destruction (1). After the bones mature, bone formation and bone resorption are under an equilibrium, thereby maintaining normal bone mass (2). Several factors might cause the destruction of this balance and the occurrence of osteoporosis, such as aging, hormone deficiency, genetic factors, *etc.* (3). As the aging population grows, osteoporosis and the related complication fragility fractures result in a high disability rate. In the United States, approximately 54 million adults over the age of 50 suffer from osteoporosis or are at risk of insufficient bone mass (4). Notably, the incidence of osteoporotic fractures is about 3 to 4 times higher than that of cardiovascular disease or cancer (5). Therefore, it is of great significance to explore its molecular mechanism and develop high-efficiency treatments for osteoporosis.

Mitochondria are the unique organelles of eukaryotes, which play an important role in maintaining homeostasis, such as metabolism, energy production, oxidative stress, and apoptosis (6, 7). Osteoporosis is a prevalent aging-related disease, which is usually accompanied by changes in metabolic processes and mitochondrial dysfunction (8, 9). And mitochondrial dysfunction will lead to the accumulation of reactive oxygen species (ROS) and induce the damage of various macromolecules such as proteins, nucleic acids, and lipids in cells (10). Besides, oxidative stress could increase the activity of osteoclasts and decrease the osteogenic potential of osteoblasts, thereby destroying bone homeostasis (11–13). All these suggest that mitochondrial dysfunction might contribute to the occurrence and development of osteoporosis.

Sirtuins (SIRTs) are a family of highly conserved NAD^+^-dependent deacetylases in mammals, which influence several metabolism processes (14–16). As a member of mitochondrial sirtuins, SIRT3 is located on chromosome 11 (Chr11p15.5) and serves a critical role in mitochondrial homeostasis, metabolic regulation, gene transcription, and genome stability (17–19). SIRT3 has been reported to be involved in a variety of aging-related diseases, such as Alzheimer’s disease, Parkinson’s disease, cardiovascular disease, and bone diseases (20, 21). A previous study showed that the senescence of bone mesenchymal stem cells (BMSCs) could lead to osteogenic damage and osteoporosis (22). And the maintenance of mitochondrial NAD^+^ levels and the expression of SIRT3 could delay the senescence of MSCs (23). Though recent evidence indicates that SIRT3 is linked to osteoporosis, the exact mechanism is still unclear. In this review, we are devoted to explaining the role and molecular mechanism of SIRT3 in osteoporosis.

## The Physiological Function of SIRT3

In the mitochondrial sirtuins, SIRT3 exhibits strong deacetylase activity, which contains a large Rossman fold domain that binds to NAD^+^ and a small domain with a zinc finger structure (24). A large amount of evidence proves that SIRT3 could regulate mitochondrial functions from many aspects, such as energy metabolism, oxidative stress, mitophagy, *etc.* (25). For example, SIRT3 could deacetylate FOXO3 under the induction of hydrogen peroxide, thereby regulating mitochondrial quality, ATP production, and clearance of defective mitochondria (26).

As one of the most basic characteristics of life, energy metabolism mainly includes the release, transfer, storage, and utilization of energy. SIRT3 could promote energy production by deacetylating the subunit proteins of the mitochondrial respiratory chain complex (27). For instance, SIRT3 could maintain intracellular metabolic balance by deacetylating ATP synthase beta (28, 29). In addition, SIRT3 could deacetylate Acetyl-CoA Synthase 2, succinate dehydrogenase and 3-hydroxy-3-methylglutaryl CoA synthase 2, thereby indirectly regulating energy production (30–32). The *in-vivo* study also confirmed that ATP production in SIRT3^-/-^ mice was approximately reduced by 50% (33). In short, SIRT3 is an important regulator of energy homeostasis.

Mitochondria are not only involved in energy metabolism, but also crucial for the production and scavenging of ROS (34). A previous study showed that SIRT3 reduced the production of ROS by deacetylating FOXO3a (35). Besides, SIRT3 could increase the activity of superoxide dismutase 2 (SOD2), and result in the reduced ROS and prevention of cell senescence (36, 37). All above evidence shows that SIRT3 serves as a key mitochondrial protein to protect cells from ROS *via* enhancing the activity of the antioxidant defense system.

Mitophagy is a kind of mitochondrial selective autophagy, which degrades damaged mitochondria in cells (38, 39). In the myocardium of SIRT3^-/-^ mice, Li et al. found that SIRT3 deficiency could significantly inhibit p53/Parkin-mediated mitophagy and promote mitochondrial dysfunction (40). The deficiency in SIRT3 could also damage the mitochondrial fission and mitophagy through FOXO3a/Parkin signaling (41). Additionally, SIRT3 acts as a key activator of mitophagy, which may be mediated by the VDAC1/Parkin pathway (42). Therefore, SIRT3 is central to the maintenance of proper mitochondrial function by regulating mitophagy through multiple pathways (43).

## The Cell Types Involved in Bone Remodeling

Bone remodeling is a dynamic process including several stages: initiation/activation stage; bone resorption stage; reversal stage; osteogenesis stage; and mineralization stage (44). This process is mainly carried out in an anatomical and functional structure called basic multicellular units, involving multiple types of cells: bone stem cells, osteocytes, osteoclasts, and osteoblasts, *etc.* (45, 46). MSCs are a group of pluripotent stem cells developed from the mesoderm and mainly exist in the bone marrow and adipose tissue (47, 48). As the common progenitor cells of osteoblasts, adipocytes, and chondrocytes, BMSCs play an important role in bone homeostasis (49). The differentiation of BMSCs is affected by many factors, such as hormones, cytokines, and mechanical factors (49). Previous studies have shown that estrogen and bone morphogenetic protein 2 (BMP2) were the signals of the osteogenic differentiation of BMSCs, and peroxisome proliferator-activated receptor γ (PPARγ) could promote the differentiation of BMSCs into adipocytes (50, 51).

Osteoblasts are vital for bone formation, which can not only differentiate into the most abundant osteocytes but also promote the mineralization of osteoid and regulate the function of osteoclasts (52–54). Osteoclasts are multinucleated cells derived from hematopoietic stem cells and are mainly responsible for bone resorption (55). It is currently clear that nuclear factor receptor activator-B (RANK)/RANK ligand (RANKL) is the main signal pathway for osteoclast differentiation and bone resorption (56, 57). In the process of osteoclast formation, bone marrow-derived macrophages differentiate into tartrate-resistant acid phosphatase^+^ (TRAP^+^) preosteoclasts under the action of the RANKL receptor activator (58). Mononuclear preosteoclast cells fuse with each other to form multinucleated mature osteoclasts. Osteoprotegerin (OPG) is secreted by a variety of cells including osteoblasts and mainly reduces bone loss by blocking the combination of RANKL and RANK (59). The OPG levels were reduced due to the aging-related decrease of osteoblasts, thereby activating osteoclast resorption and causing osteoporosis (60). Interestingly, osteocytes, as the protagonist of bone formation, are also the main source of RANKL, thereby promoting the occurrence of osteoclasts (61). In the dynamic process of bone remodeling, all participating cells might interact or restrict each other to achieve bone homeostasis.

## The Role of SIRT3 in Bone Remodeling and Osteoporosis

**Figure 1:**
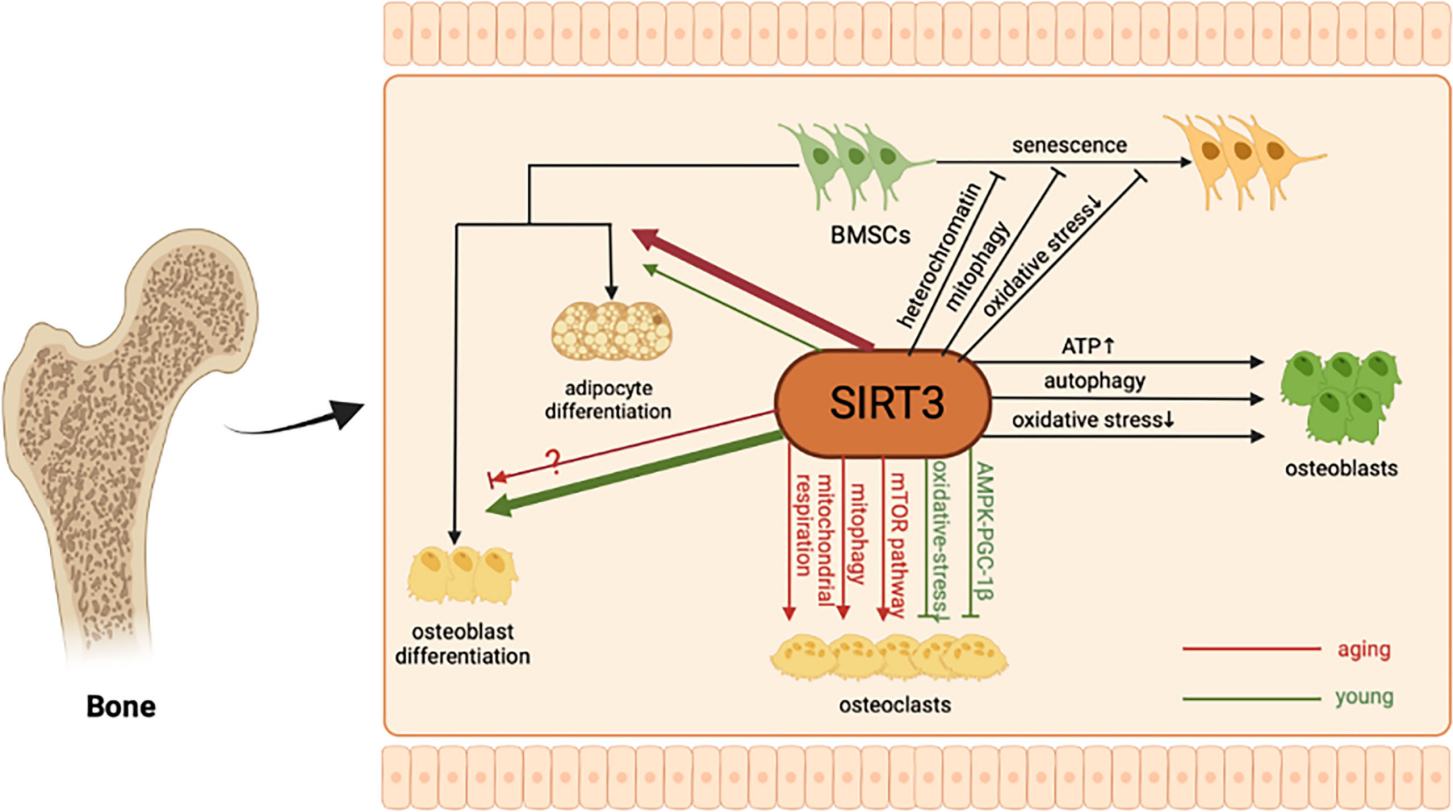
The molecular mechanism of SIRT3 in bone remodeling. As a major mitochondrial protein deacetylase, SIRT3 can alleviate BMSCs senescence by regulating oxidative stress, mitophagy, and stabilizing heterochromatin. In addition, SIRT3 could protect osteoblasts and promote bone formation through multiple pathways. Regarding BMSCs differentiation and osteoclastogenesis, the regulatory role of SIRT3 may be age-related.

Osteoporosis is a chronic disease caused by the imbalance of bone formation and bone resorption (62). SIRT3 has been demonstrated to play an important role in bone remodeling. Next, we will explain how SIRT3 participates in bone remodeling and osteoporosis from different aspects, including the senescence and differentiation of BMSCs, differentiation of osteoblasts, osteoclastogenesis, and changes in bone mass (**Figure 1**).

### The Role of SIRT3 in the Senescence of BMSCs

BMSCs are characterized by self-renewal and multi-differentiation potential (63). And the senescence and aberrant differentiation of BMSCs are related to a variety of pathophysiological processes, including osteoporosis (64, 65). The increased oxidative stress is a major feature of senescent BMSCs (66, 67). SIRT3 could reduce oxidative stress-caused BMSCs apoptosis *via* activating manganese superoxide dismutase (MnSOD, an alias for SOD2) and catalase (68). In the BMSCs models of natural senescence and H_2_O_2_-induced premature senescence, the expression of SIRT3 was significantly reduced, which was related to the decrease of antioxidant capacity and the aggravation of DNA damage (69). SIRT3 supplementation could alleviate BMSCs senescence by reducing ROS-induced damage, and enhancing the expression and activity of SOD2. Besides, SIRT3 positively regulates catalase and SOD2 by translocating FOXO3a into the nucleus, thereby protecting aged donor BMSCs from oxidative damage (70, 71). All the above evidence indicates that SIRT3 could regulate the senescence of BMSCs through oxidative stress-related pathways.

Abnormal mitophagy could damage mitochondrial quality and function, and play a key role in stem cell maintenance and differentiation (39, 72–74). Guo et al. found that advanced glycation end products (AGEs) could destroy mitochondria function and mitophagy, eventually leading to cell senescence (75). Interestingly, the silencing of SIRT3 can further strengthen the effect of AGEs, and the overexpression of SIRT3 can significantly reduce the occurrence of BMSCs aging and osteoporosis. Regulation of mitophagy was another mechanism for SIRT3 to reduce the senescence of BMSCs and senile osteoporosis.

In addition to mitochondrial homeostasis, stem cell aging is also accompanied by various epigenetic changes, including abnormal DNA methylation, histone modification, and disorganized heterochromatin (76, 77). Previous studies have shown that SIRT3 could take a deacetylation effect in mitochondria, thereby regulating the formation of epigenetic regulators acetyl-Coenzyme a and β-hydroxybutyric acid (78, 79). Additionally, heterochromatin is an important epigenetic driving factor in the regulation of aging (80, 81). Diao et al. found that SIRT3 interacted with nuclear lamina proteins and heterochromatin-related proteins to consolidate heterochromatin, which partly explained the mechanism of SIRT3 in preventing the senescence of BMSCs (82).

In summary, SIRT3, as a longevity gene, could not only maintain the mitochondrial function in the mitochondria but also stabilize the heterochromatin in the nucleus, so as to delay the senescence of BMSCs.

### The Role of SIRT3 in the Differentiation of BMSCs

BMSCs have great potential for bone remodeling due to their osteogenic differentiation ability. As aging progresses, adipogenic differentiation of BMSCs increases and osteogenic differentiation weakens, causing abnormal bone metabolism (83, 84). The SIRT3 deletion reduces the differentiation of BMSCs into adipocytes and osteoblasts, while overexpression of SIRT3 enhances the differentiation ability of young BMSCs (passage 3) and aging BMSCs (passage 7) (85). Ho et al. found that SIRT3 overexpression resulted in more adipocytes in the bone marrow niche and the decreased bone mass in aging male mice (86). However, the regulation role of SIRT3 in bone was not observed in young (6-month-old) and female mice. Furthermore, BMSCs derived from aging mice overexpressing SIRT3 showed a stronger ability to differentiate into adipocytes compared with the control group, and osteoblastogenesis was suppressed (86). Interestingly, such results are inconsistent with previous research findings. Instead of protecting bone loss, SIRT3 has deleterious effects on osteogenic differentiation of BMSCs. It suggests that SIRT3 plays a complex regulatory role in the differentiation of BMSCs and the maintenance of bone homeostasis, which may be related to age and gender, but its specific mechanism need to be further explored.

### The Role of SIRT3 in Osteoblasts

Osteoporosis is associated with increased levels of oxidative stress in osteoblasts, which may be a key component of the pathophysiology of bone loss (87). Li et al. found that nicotine could induce mitochondrial oxidative stress and mitochondrial DNA (mtDNA) damage in osteoblasts, ultimately leading to osteoporosis. Mechanically, nicotine can reduce the SIRT3 level, thereby significantly reducing the deacetylation level and activity of SOD2 in osteoblasts (88). In mouse pre-osteoblastic MC3T3-E1 cells, SIRT3 deletion could downregulate mitochondrial function and biogenesis through the PGC-1α/SOD2 signaling pathway, leading to impairment of osteogenesis (89). More importantly, SIRT3 deficiency could contribute to the impaired osteoblast function, bone loss and osteoporosis in SIRT3^−/−^ mice. Both *in-vivo* and *in-vitro* experiments have proved that the SIRT3 could support increased ATP production, robust mitochondrial biogenesis, and osteoblast differentiation *via* deacetylating SOD2 (90).

Titanium is an important material for prostheses and stents for bone reconstruction, which could increase the durability of mechanical damage (91). With the development of nano-toxicology, nano-materials have been confirmed to be cytotoxic (92). For instance, titanium can cause osteoblast damage through autophagy and excessive mitochondrion-derived ROS (mROS). SIRT3 could reduce the acetylation of SOD2, the production of titanium-induced mROS and the expression level of LC3, thereby improving the viability of osteoblasts (93). Therefore, the SIRT3 and SOD2 may form an important regulatory network to protect osteoblasts against the cytotoxic of TiO2NPs (94). Besides, upregulation of SIRT3 significantly attenuates the titanium particle-induced inhibition of osteogenesis by inhibiting the NLRP3 inflammasome (95). To sum up, SIRT3 plays a protective role in bone formation and maintaining bone homeostasis.

### The Role of SIRT3 in Osteoclasts

Osteoclasts, the protagonist of bone resorption, mainly resorb the mineralized bone matrix to maintain bone and mineral homeostasis (96, 97). As an indispensable element in various cell signal transduction, ROS acts as the second messenger in the process of osteoclast differentiation and activation induced by RANKL (98). Haemin et al. found that SIRT3 downregulation enhanced osteoclasts formation and RANKL-induced bone loss in five-week-old female ICR mice. Mechanically, SIRT3 could enhance SOD2 activity through deacetylation of lysine 68 and reduce intracellular ROS level, thereby inhibiting osteoclast differentiation (98). What’s more, SIRT3 could inhibit the osteoclast differentiation by regulating AMPK-PGC-1β pathway (99). And the decreased bone mass was observed in the young SIRT3^−/−^ mice (eight-week-old), which was mainly caused by increased osteoclastogenesis (99). Therefore, SIRT3 might serve as a negative regulator in osteoclast differentiation and bone mass at a young age.

On the contrary, SIRT3 could promote osteoclastogenesis and bone loss by activating the mechanistic target of rapamycin (mTOR) pathway in aging transgenic overexpressing SIRT3 mice (13-month-old) (86). Additional researchers found that deletion of SIRT3 had no effect on bone in young mice, but attenuated age-related bone loss in 16-month-old mice. Loss of SIRT3 impaired bone resorption by reducing the mitochondrial respiration and mitophagy of osteoclasts, but had no effect on osteoclast number (100). It seems that elevated SIRT3 may promote bone loss in old age, partly due to an imbalance in bone resorption.

Aging and sex steroid deficiency are two common causes of osteoporosis (101). Therefore, in addition to aging models, the researchers also constructed estrogen deficiency-related animal models to study the role of SIRT3 in osteoporosis (100). In 5-month-old ovariectomized SIRT3^-/-^ mice, remission of ovariectomy-induced cortical bone loss can be observed, accompanied by a decreased bone resorption instead of an increase in bone formation (100). How estrogen directly or indirectly affects the role of SIRT3 in mitochondria as well as bone homeostasis needs to be dissected.

In addition to aging and sex steroid-related bone loss, SIRT3 also takes part in the pathogenesis of ionizing radiation exposure-induced osteoporosis. Ionizing radiation could increase the expression and enzymatic activity of SIRT3 in osteoclasts and cause osteoclast differentiation and bone loss in young adult male mice (102). Nevertheless, the study did not explore the role of SIRT3 in the response of female and elderly mice to ionizing radiation.

Moreover, titanium has a side effect on the formation and function of osteoclasts. Surprisingly, in the murine model of osteolysis induced by titanium particles, inhibition of SIRT3 could prevent titanium particle-induced bone resorption and osteoclast formation by inhibiting ERK and JNK signals (103). Therefore, SIRT3 could regulate bone resorption to alleviate the cytotoxicity of titanium. It’s still required to elucidate how SIRT3 maintains the balance between bone resorption and bone formation.

Taken together, SIRT3 might play an important regulatory role in bone formation and bone resorption through multiple pathways. It seems that SIRT3 acts to promote BMSCs osteogenic differentiation and inhibit osteoclast differentiation to stabilize bone mass in young age, while in aging stage, SIRT3 may promote adipogenic differentiation of BMSCs, osteoclast differentiation and bone resorption, eventually leading to bone loss. However, it is still unknown when and how this regulatory effect is switched. Second, how SIRT3 stabilizes in bone formation and resorption remains unexplained. Indeed, downregulation of SIRT3 inhibited osteoclast differentiation in aging, accompanied by a small decrease in osteoblast production (100). Therefore, various pathological or health conditions and osteoporosis caused by different reasons should be considered to explain these inconsistent results and the role of SIRT3 in osteoporosis.

## Sirt3 as a Potential Target for the Treatment of Osteoporosis

Accumulating evidence has indicated that intervention for SIRT3 could improve osteoporosis, showing its potential as a treatment strategy. Exogenous supplementation or overexpression of SIRT3 could alleviate bone loss and osteoporosis (75, 104). For example, the overexpression of SIRT3 by intravenous injection of recombinant adeno-associated virus 9 carrying SIRT3 plasmid (AAV9-SIRT3) could significantly reduce the occurrence of senile osteoporosis in the mouse model (75). Mild hypoxia pretreatment combined with curcumin could improve the mitochondrial function of BMSCs through PGC-1α/SIRT3/HIF-1α signal, and significantly increase cell survival (105).

Zoledronic acid is currently a common drug for the treatment of osteoporosis (106). It could enhance osteogenic differentiation of BMSCs, inhibit osteoclast activity and induce osteoclast apoptosis, thereby alleviating osteoporosis (107, 108). Recent research showed that zoledronic acid might inhibit oxidative stress through the SIRT3/SOD2 pathway to accelerate BMSCs osteogenesis and alleviate the progression of osteoporosis (109).

As an indolamine hormone and a potent free radical scavenger, melatonin could improve osteoporosis through promoting osteoblast differentiation and bone formation (110–112). Mechanism study showed that melatonin could improve mitochondrial oxidative stress through SIRT3/SOD2 signaling pathway, thereby promoting bone formation and improving bone mass loss (113, 114). Also, melatonin could inhibit oxidative damage in preosteoblasts and promote osteogenesis by activating SIRT1, which then regulates the expression of p66Shc and SIRT3 (115).

Resveratrol is a natural polyphenol found in red wine, which can inhibit osteoclast differentiation (116). Matsuda et al. found that resveratrol could alleviate dexamethasone-induced inhibition of BMP2 and OPG expression and mitochondrial dysfunction (117). Moreover, resveratrol might stimulate the SIRT3/PGC-1α/SOD2 axis by activating AMPK, thereby improving mitochondrial dysfunction and protecting osteoblasts against dexamethasone-induced cytotoxicity.

Metformin, a traditional antidiabetic drug, has been shown to have multiple efficacies in the treatment of tumors and aging (118, 119). Metformin may attenuate diabetes-related osteoporosis by improving the hyperglycemic microenvironment (120). And metformin could upregulate SIRT3 expression *via* PI3K/AKT pathway and reverse H_2_O_2_-induced osteoblast apoptosis (121).

Nevertheless, in addition to alleviating the aging of BMSCs and promoting osteogenic differentiation, it was confirmed that SIRT3 could promote osteoclast differentiation and bone resorption in aging male mice. The SIRT3 inhibitor LC-0296 was shown to increase bone mass in aging mice (100). Besides, intervention of osteoclast progenitors in 16-month-old female C57BL/6 mice with LC-0296 reduced osteoclast formation, which was consistent with the results in aged SIRT3^-/-^ mice. From the above-mentioned mechanism of SIRT3 in bone remodeling, the positive or negative effects of SIRT3 may be related to age and gender. In the same way, the use of upregulation and downregulation of SIRT3 to treat osteoporosis needs to be evaluated according to different situations. In the young period, SIRT3 should be upregulated to promote osteogenesis, while in the aging period, SIRT3 should be downregulated to inhibit bone resorption and alleviate osteoporosis. Of course, more animal experiments and clinical studies need to be carried out to confirm this speculation.

## Conclusion

Osteoporosis is a common senile disease caused by a variety of factors (112). Since SIRT3 contains an N-terminal mitochondrial signal sequence, it is mainly located in the mitochondria. And SIRT3 has important implications for aging and diseases by regulating mitochondrial biology (122). In this review, SIRT3 could participate in several physiological processes including the senescence and differentiation of BMSCs, and osteoclastogenesis, thereby modulating osteoporosis. In general, SIRT3 alleviates the senescence of BMSCs by regulating oxidative stress, mitophagy, and stabilizing heterochromatin. As for the differentiation of BMSCs, osteoblastogenesis and osteoclastogenesis, the role and mechanism of SIRT3 vary with different conditions, especially age.

In conclusion, SIRT3 plays a vital role in maintaining the balance of bone formation and bone resorption. However, regarding the role of SIRT3 in bone metabolism under physiological and pathological conditions, there is still much to study.

## Author Contributions

SW contributed to the conception, design, and final approval of the submitted version. SH contributed to completing the Figure, writing the paper. The authors have read and approved the final manuscript.

## Funding

The research was supported by the grant from: National Natural Science Foundation of China (81900441), Natural Science Foundation of Zhejiang Province (LQ19H020002), Zhejiang Provincial Program for Medicine and Health (2022KY446), Social Development Science and Technology Foundation of Taizhou (21ywb115, 21ywb118, 20ywb143), Social Development Science and Technology Foundation of Wenling (2020S0180083, 2021S00156, 2021S00197, 2020S0180127).

## Conflict of Interest

The authors declare that the research was conducted in the absence of any commercial or financial relationships that could be construed as a potential conflict of interest.

## Publisher’s Note

All claims expressed in this article are solely those of the authors and do not necessarily represent those of their affiliated organizations, or those of the publisher, the editors and the reviewers. Any product that may be evaluated in this article, or claim that may be made by its manufacturer, is not guaranteed or endorsed by the publisher.
